# Metabolic and Functional Interactions of H_2_S and Sucrose in Maize Thermotolerance through Redox Homeodynamics

**DOI:** 10.3390/ijms25126598

**Published:** 2024-06-15

**Authors:** Xiao-Er Li, Hong-Dan Zhou, Zhong-Guang Li

**Affiliations:** 1 School of Life Sciences, Yunnan Normal University, Kunming 650092, China; 2 Engineering Research Center of Sustainable Development and Utilization of Biomass Energy, Ministry of Education, Kunming 650092, China; 3 Key Laboratory of Biomass Energy and Environmental Biotechnology, Yunnan Province, Yunnan Normal University, Kunming 650092, China

**Keywords:** hydrogen sulfide, maize seedlings, redox homeodynamics, sucrose signaling, thermotolerance

## Abstract

Hydrogen sulfide (H_2_S) is a novel gasotransmitter. Sucrose (SUC) is a source of cellular energy and a signaling molecule. Maize is the third most common food crop worldwide. However, the interaction of H_2_S and SUC in maize thermotolerance is not widely known. In this study, using maize seedlings as materials, the metabolic and functional interactions of H_2_S and SUC in maize thermotolerance were investigated. The data show that under heat stress, the survival rate and tissue viability were increased by exogenous SUC, while the malondialdehyde content and electrolyte leakage were reduced by SUC, indicating SUC could increase maize thermotolerance. Also, SUC-promoted thermotolerance was enhanced by H_2_S, while separately weakened by an inhibitor (propargylglycine) and a scavenger (hypotaurine) of H_2_S and a SUC-transport inhibitor (N-ethylmaleimide), suggesting the interaction of H_2_S and SUC in the development of maize thermotolerance. To establish the underlying mechanism of H_2_S–SUC interaction-promoted thermotolerance, redox parameters in mesocotyls of maize seedlings were measured before and after heat stress. The data indicate that the activity and gene expression of H_2_S-metabolizing enzymes were up-regulated by SUC, whereas H_2_S had no significant effect on the activity and gene expression of SUC-metabolizing enzymes. In addition, the activity and gene expression of catalase, glutathione reductase, ascorbate peroxidase, peroxidase, dehydroascorbate reductase, monodehydroascorbate reductase, and superoxide dismutase were reinforced by H_2_S, SUC, and their combination under non-heat and heat conditions to varying degrees. Similarly, the content of ascorbic acid, flavone, carotenoid, and polyphenol was increased by H_2_S, SUC, and their combination, whereas the production of superoxide radicals and the hydrogen peroxide level were impaired by these treatments to different extents. These results imply that the metabolic and functional interactions of H_2_S and sucrose signaling exist in the formation of maize thermotolerance through redox homeodynamics. This finding lays the theoretical basis for developing climate-resistant maize crops and improving food security.

## 1. Introduction

Hydrogen sulfide (H_2_S) is a novel gasotransmitter which participates in many physiological processes from seed germination to organ senescence [1,2]. H_2_S, similar to other signaling molecules, has a dual role as a signaling molecule and cytotoxin in a dose-dependent manner [2,3]. Therefore, the action of H_2_S is strictly controlled by its homeostasis system, which includes enzymatic and non-enzymatic pathways. In plants, the enzymatic pathways for H_2_S biosynthesis involve L-cysteine desulfhydrase (LCD), D-cysteine desulfhydrase (DCD), O-acetyl-serine (thiol) lyase (OAS-TL), carbonic anhydrase (CA), mercaptopyruvate sulfur transferase (MST), sulfite reductase (SiR), β-cyanoalanine synthase (CAS), and nitrogenase Fe-S cluster-like (NFS) [2,4]. These enzymes use L-/D-cysteine, carbonyl sulfide, and sulfite as substances to synthesize H_2_S. In non-enzymatic pathways for H_2_S production, H_2_S can be released from organic and inorganic compounds, such as persulfides and polysulfides, under the influence of reducing agents like glutathione (GSH) [2,5]. In recent years, substantial studies have reported that H_2_S as a signaling molecule modulates seed germination, stomatal movement, floral induction, organ senescence, and plant stress response [2,4,5,6]. Our studies also found that as a signaling molecule, H_2_S was able to increase maize thermotolerance, which is implicated in the signaling crosstalk of H_2_S with calcium, nitric oxide, methylglyocal, and abscisic acid [2,7,8]. However, whether H_2_S-promoted maize thermotolerance is related to sucrose signaling remains unclear.

Sucrose (SUC) is a disaccharide and is a source of cellular energy and a signaling molecule, regulating the whole life processes [9,10,11]. In general, SUC can exert its signaling role in plants through multiple pathways, which are involved in hexose kinase-dependent and hexose kinase-independent signaling [12,13,14]. Also, SUC, as a non-structural sugar, has a high solubility, exhibiting multiple physiological functions, such as an energy source, a nutrient, an osmotic adjustment substance, and a reactive oxygen species (ROS) scavenger [15,16,17]. In addition, SUC is a short- and long-distance signaling molecule which regulates seed germination, seedling establishment, and plant growth and development, as well as the response and adaptation to environmental stresses [1,13,14,16,17]. In potato and rice plants, SUC treatment could boost thermotolerance by activating the ROS-scavenging system [18,19], but the relationship between SUC-promoted thermotolerance and H_2_S signaling is not widely known. Also, in the model plant Arabidopsis, Aroca et al. [20], using the proteomics approach, found that at least 5% (approximately 2330 proteins) of the entire Arabidopsis proteome was able to be persulfidated. These persulfidated proteins are mainly involved in the enzymes related to SUC and starch metabolism (mainly glycolysis and the Calvin cycle) [20]. This study hints for the first time at the underlying crosstalk of SUC and H_2_S in plants.

Maize not only is a food, feed, and energy crop, but also a novel model plant, whose genome was sequenced in 2009 [21]. Due to its multiple roles, global maize production and consumption is increasing year by year, with maize becoming a leading cereal crop [22]. Although maize is a temperature-loving crop, its growth and development, especially in the seedling stage, are sensitive to heat stress [7,23,24,25,26]. A 1 °C increase in average global temperature could lead to a reduction in maize yield by 7.4% [23,24]. With the exacerbation of global warming, heat stress has become a major stress factor that limits maize growth, development, and reproduction [27,28]. The underlying mechanism of maize thermotolerance has to be settled urgently in order to develop climate-resilient maize crops for sustainable agriculture and food security. Therefore, in this paper, using maize seedlings as materials, the metabolic and functional interactions of H_2_S and SUC in maize thermotolerance were investigated. The purpose of this paper is to establish the interaction of H_2_S and SUC in the development of maize thermotolerance via redox homeodynamics.

## 2. Results

### 2.1. H_2_S and SUC Upraises Thermotolerance

**Figure 1:**
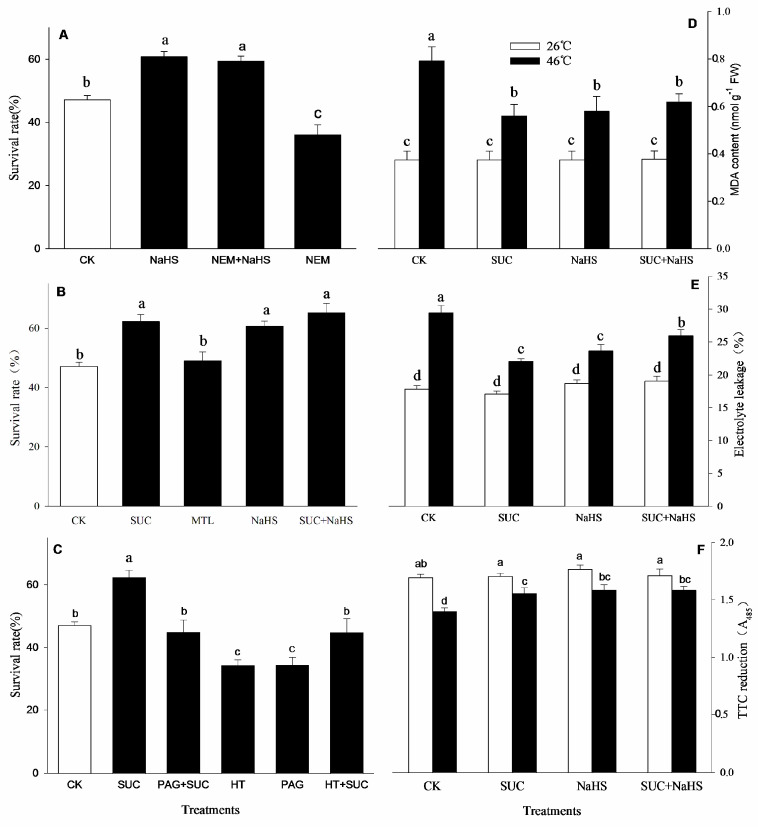
Effect of pretreatment with NaHS, sucrose (SUC), mannitol (MTL), N-ethylmaleimide (NEM), propargylglycine (PAG), and hydroxylamine (HT) alone or in combination on the survival rate ((**A**–**C**), %), malondialdehyde (MDA, (**D**)), electrolyte leakage (**E**), and tissue viability (**F**) of maize seedlings under heat stress conditions. Significance between treatments was evaluated using the Duncan multiple-range test, and the data in the figures denote means ± standard errors (SEs, *n* = 4), while the same and different letters on the bars indicate no significant and significant differences, respectively.

After heat stress and recovery, the survival rate of the maize seedlings was calculated, and the results are shown in Figure 1. Compared to the control, the survival rate of the seedlings irrigated with NaHS, SUC, and their combination after heat stress was significantly increased (Figure 1A,B), while MTL treatment had no significant effect on the survival rate (Figure 1B). Also, the survival rate was markedly improved by NEM combined with NaHS (Figure 1A), but NEM alone worsened the survival rate (Figure 1A) compared with the control. In addition, the increased SR caused by SUC was separately abolished by PAG + SUC and HT + SUC, and was deteriorated by PAG and HT alone (Figure 1C). Similarly, compared with the control, under non-heat stress conditions (at 26 °C), NaHS and SUC alone or in combination had no significant effect on MDA accumulation, electrolyte leakage, and tissue viability (TTC reduction) in maize seedlings (Figure 1D–F). Under heat stress conditions (at 46 °C), an increase in the MDA level and electrolyte leakage as well as a decrease in tissue viability in maize seedlings were alleviated by NaHS and SUC alone or in combination compared to the control (Figure 1D–F).

### 2.2. SUC Upraises H_2_S Level

**Figure 2:**
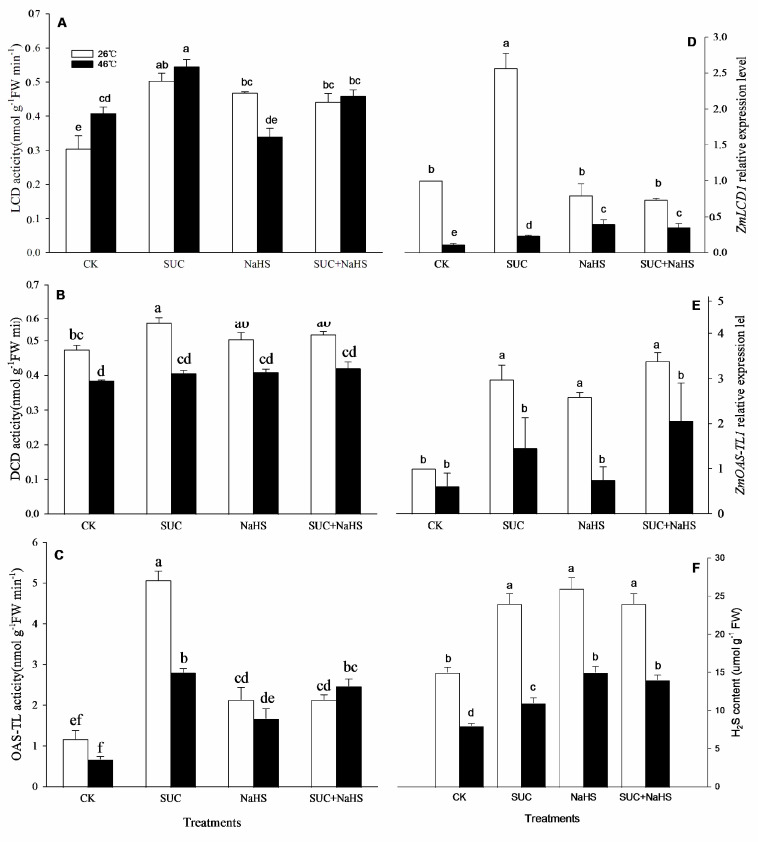
Effect of pretreatment with sucrose (SUC) and NaHS alone or in combination on L-cysteine desulfhydrase (LCD, (**A**)), D-cysteine desulfhydrase (DCD, (**B**)), and O-acetyl-serine (thiol) lyase (OAS-TL, (**C**)) activities, the gene expression of *ZmLCD1* (**D**) and *ZmOAS-TL* (**E**), and the endogenous H_2_S content (**F**) in maize seedlings under non-heat and heat stress conditions. Significance between treatments was evaluated using the Duncan multiple-range test, and the data in the figures denote means ± standard errors (SEs, *n* = 6), while the same and different letters on the bars indicate no significant and significant differences, respectively.

To study the possible effect of SUC on H_2_S level, endogenous H_2_S and its metabolizing enzymes in maize seedlings were analyzed. The results shown in Figure 2, under non-heat stress conditions, compared with the control, LCD, DCD, and OAS-TL activities in maize seedlings were enhanced by SUC (Figure 2A–C). Also, LCD and OAS-TL activities were increased by NaHS alone or combined with SUC, which had no significant effect on DCD activity (Figure 2A–C). Similarly, the gene expression of *ZmLCD1* and *ZmOAS-TL1* in maize seedlings was up-regulated by SUC, while *ZmOAS-TL1* expression was also enhanced by NaHS alone or combined with SUC, while there was no significant impact on *ZmLCD1* expression compared with the control (Figure 2D,E). In addition, compared with the control, the endogenous H_2_S level in maize seedlings was obviously enhanced by SUC and NaHS alone or in combination (Figure 2F).

Similarly, under heat stress conditions, compared to the control, LCD and OAS-TL activities in maize seedlings were enhanced by SUC, NaHS, and SUC + NaHS, while they did not have a significant impact on DCD activity (Figure 2A–C). Moreover, the gene expression of *ZmLCD1* in maize seedlings was significantly up-regulated by SUC, NaHS, and SUC + NaHS, while they did not have a significant effect on *ZmOAS-TL1* expression (Figure 2D,E). Analogously, compared to the control, the endogenous H_2_S level in maize seedlings was markedly increased by SUC, NaHS, and SUC + NaHS (Figure 2F).

### 2.3. H_2_S Modulates SUC Level

**Figure 3:**
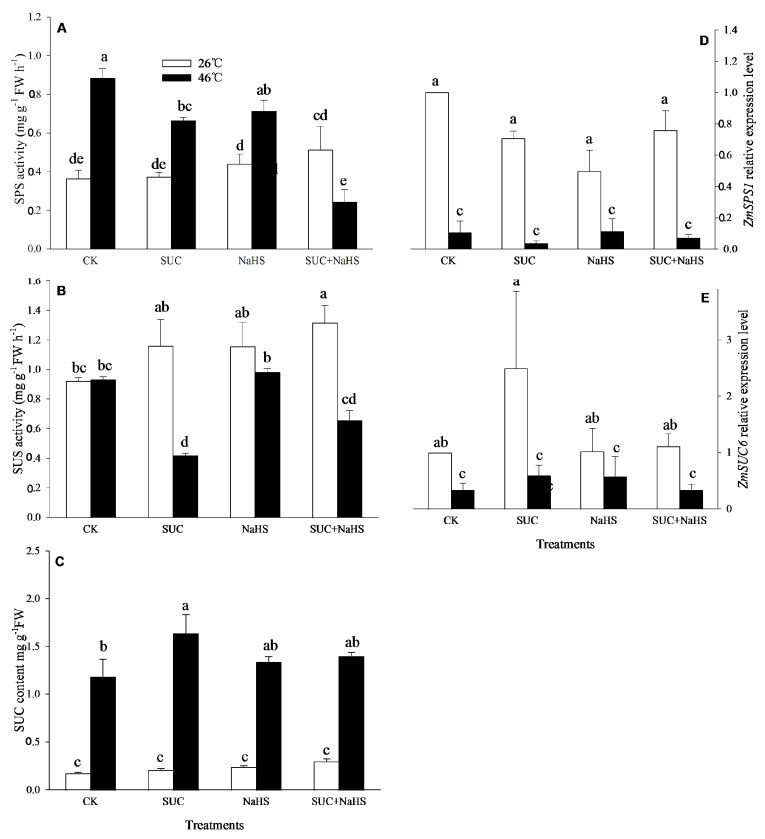
Effect of pretreatment with sucrose (SUC) and NaHS alone or in combination on sucrose-P synthase (SPS, (**A**)) and sucrose synthase (SUS, (**B**)) activities, SUC content (**C**), and the gene expression of *ZmSPS1* (**D**) and *ZmSUS6* (**E**) in maize seedlings under non-heat and heat stress conditions. Significance between treatments was evaluated using the Duncan multiple-range test, and the data in the figures denote means ± standard errors (SEs, *n* = 5), while the same and different letters on the bars indicate no significant and significant difference, respectively.

To further explore the effect of H_2_S on the endogenous SUC level, SUC and its metabolizing enzymes in maize seedlings were estimated. The results show that, under non-heat stress conditions, SUS activity in maize seedlings was significant increased by SUC + NaHS, while no significant impact on SPS activity was observed compared with the control (Figure 3A,B). Also, compared to the control, a significant increase in SPS activity and SUC content in maize seedlings caused by SUC and NaHS alone or in combination was not observed (Figure 3A,C). Similarly, SUC and NaHS alone had no significant effect on SPS activity (Figure 3B). Moreover, SUN and NaHS alone or in combination had no significant impact on the gene expression of *ZmSPS1* and *ZmSUS6* in maize seedlings compared with the control (Figure 3D,E).

Also, under heat stress conditions, compared with the control, SPS and SUS activities in maize seedlings were markedly reduced by SUC and SUC + NaHS, while NaHS alone had no significant effect on enzyme activity (Figure 3A,B). Similar to non-heat stress conditions, compared with the control, a significant up-regulation of gene expression of *ZmSPS1* and *ZmSUS6* in maize seedlings was not observed (Figure 3D,E). In addition, the endogenous SUC content in maize seedlings was enhanced by exogenous SUS, whereas NaHS and NaHS + SUC did not have a significant effect on the endogenous SUC level compared with the control (Figure 3C).

### 2.4. H_2_S–SUC Interaction Enhances Antioxidant Capacity

**Figure 4:**
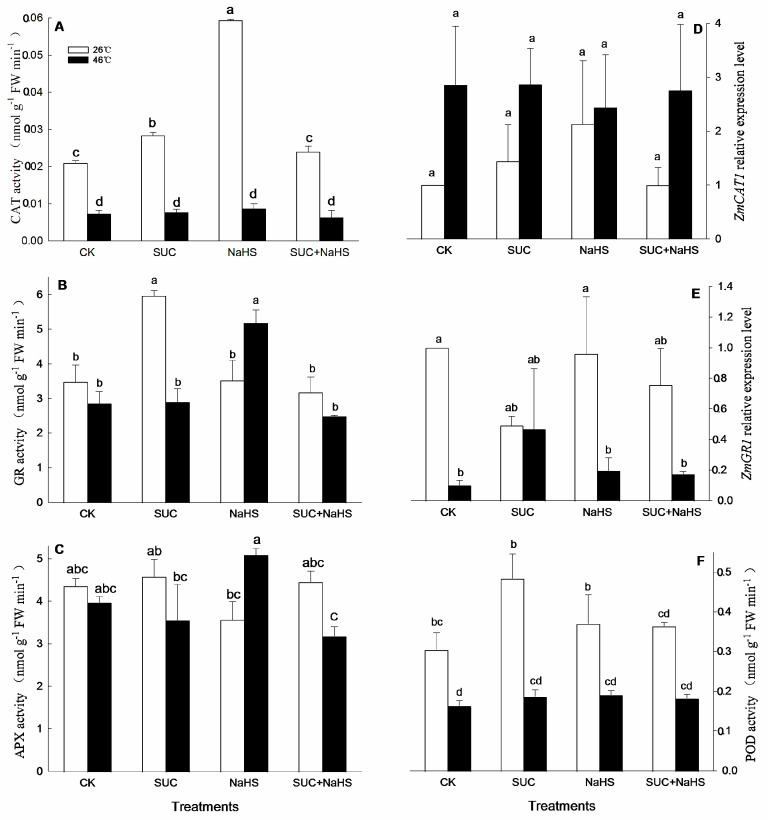
Effect of pretreatment with sucrose (SUC) and NaHS alone or in combination on catalase (CAT, (**A**)), glutathione reductase (GR, (**B**)), ascorbate peroxidase (APX, (**C**)), and peroxidase (POD, **F**) activities and the gene expression of *ZmCAT* (**D**) and *ZmGR1* (**E**) in maize seedlings under non-heat and heat stress conditions. Significance between treatments was evaluated using the Duncan multiple-range test, and the data in the figures denote means ± standard errors (SEs, *n* = 5), while the same and different letters on the bars indicate no significant and significant difference, respectively.

**Figure 5:**
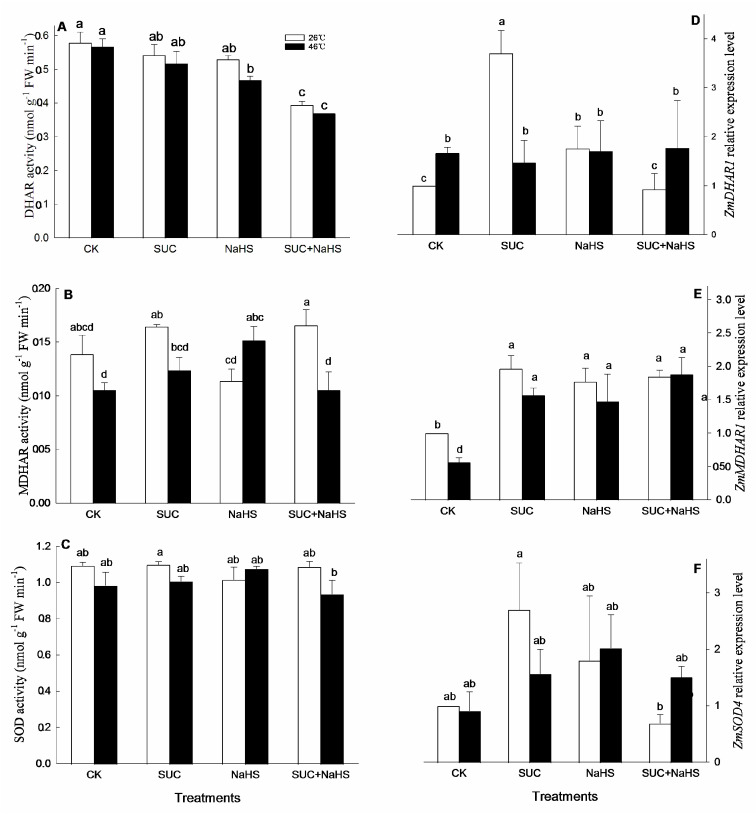
Effect of pretreatment with sucrose (SUC) and NaHS alone or in combination on dehydroascorbate reductase (DHAR, (**A**)), monodehydroascorbate reductase (MDHAR, (**B**)), and superoxide dismutase (SOD, (**C**)) activities and gene expression of *ZmDHAR1* (**D**), *ZmMDHAR1* (**E**), and *ZmSOD4* (**F**) in maize seedlings under non-heat and heat stress conditions. Significance between treatments was evaluated using the Duncan multiple-range test, and the data in the figures denote means ± standard errors (SEs, *n* = 4), while the same and different letters on the bars indicate no significant and significant difference, respectively.

To further illustrate the underlying mechanism of H_2_S–SUC interaction-promoted maize thermotolerance, cellular redox parameters in maize seedlings were assessed. Under non-heat stress conditions, compared with the control, CAT and GR activities in maize seedlings were significant increased by exogenous SUC (Figure 4A,B). Similarly, CAT activity in maize seedlings was significantly enhanced by NaSH (Figure 4A), while SUN and NaHS alone or in combination had no significant effect on APX, POD, DHAR, MDHAR, and SOD activities in maize seedlings (Figure 4C and Figure 5A–C). Also, compared with the control, the gene expression of *ZmDHAR1* and *ZmMDHAR1* in maize seedlings was significant up-regulated by SUN, NaHS, and SUC + NaHS (Figure 5D,E), while they had no significant effect on the expression of *ZmCAT1*, *ZmGR1*, and *ZmSOD4* in maize seedlings (Figure 4D,E and Figure 5F).

Similarly, under heat stress, compared with the control, GR and DHAR activities in maize seedlings were increased by NaHS (Figure 4A and Figure 5B), while it did not have a significant effect on CAT, APX, POD, and SOD activities (Figure 4A,C,F and Figure 5C), but reduced DHAR activity (Figure 5A). Also, SUC alone or combined with NaHS had no significant impact on CAT, GR, APX, POD, DHAR, MDHAR, and SOD activities in maize seedlings compared with the control (Figure 4A–C,F and Figure 5B,C), except for combined treatment reducing DHAR activity (Figure 5A). Similarly, a gene expression of *ZmMDHAR1* in maize seedlings was up-regulated by SUC, NaHS, and SUC + NaHS, while they did not have a significant influence on *ZmCAT1*, *ZmGR1*, *ZmDHAR1*, and *ZmSOD4* in maize seedlings compared with the control (Figure 4D,E and Figure 5D,F).

**Figure 6:**
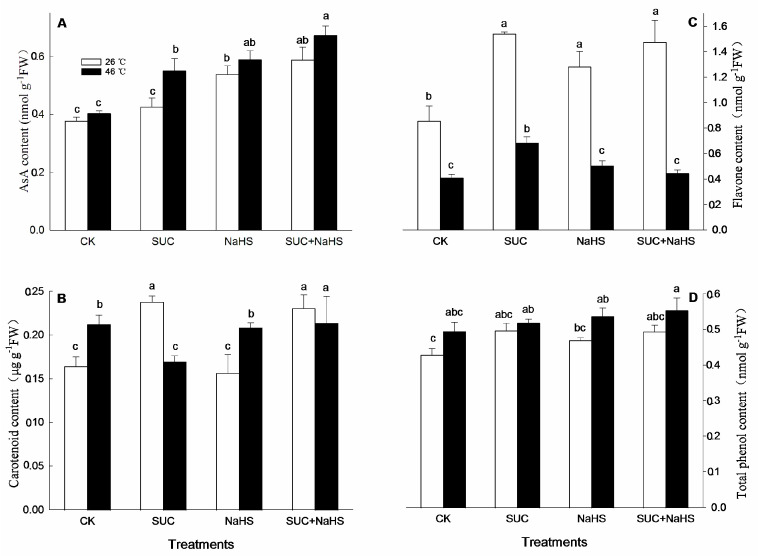
Effect of pretreatment with sucrose (SUC) and NaHS alone or in combination on ascorbic acid (AsA, (**A**)), carotenoid (**B**), flavone (**C**), and total phenol (**D**) contents in maize seedlings under non-heat and heat stress conditions. Significance between treatments was evaluated using the Duncan multiple-range test, and the data in the figures denote means ± standard errors (SEs, *n* = 4), while the same and different letters on the bars indicate no significant and significant difference, respectively.

For antioxidant and secondary metabolites, under non-heat stress conditions, compared with the control, AsA and flavone contents in maize seedlings were enhanced by NaHS and NaHS + SUC (Figure 6A,D), while they had no significant influence on total phenol content (Figure 6D). Similarly, compared to the control, flavone and carotenoid contents in maize seedlings were markedly increased by SUC (Figure 6B,D), and the later level was also enhanced by SUC + NaHS (Figure 6B). Also, SUC alone had no significant effect on AsA and total phenol contents in maize seedlings (Figure 6A,D), while the effect of NaHS on carotenoid and total phenol contents indicated similar results (Figure 6B,D).

Under heat stress conditions, compared to the control, the AsA content in maize seedlings was significant enhanced by SUC and NaHS alone or in combination (Figure 6A), while they had no significant effect on total phenol content, but maintained a high total phenol level (Figure 6A). Analogously, flavone and carotenoid contents in maize seedlings were obviously increased by SUC and SUC + NaHS, respectively (Figure 6B,D). However, compared to the control, a significant increase in flavone level in maize seedlings treated with NaHS and SUC + NaHS was not noted (Figure 6D), while similar results were recorded in maize seedlings treated with NaHS, but SUC reduced the carotenoid level (Figure 6B).

### 2.5. H_2_S–SUC Interaction Regulates ROS Level

**Figure 7:**
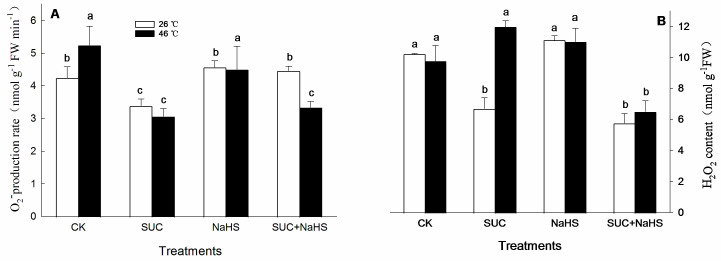
Effect of pretreatment with sucrose (SUC) and NaHS alone or in combination on the production of superoxide radical (O_2_^•−^, **A**) and hydrogen peroxide level (**B**) in maize seedlings under non-heat and heat stress conditions. Significance between treatments was evaluated using the Duncan multiple-range test, and the data in the figures denote means ± standard errors (SEs, *n* = 6), while the same and different letters on the bars indicate no significant and significant difference, respectively.

As stated above, H_2_S–SUC interaction was able to enhance cellular antioxidant capacity in maize seedlings under both non-heat and heat stress conditions (Figure 4, Figure 5 and Figure 6). To further establish the influence of H_2_S–SUC interaction on the production of superoxide radicals (O_2_^•−^) and the hydrogen peroxide (H_2_O_2_) level in maize seedlings under non-heat and heat stress conditions, their production and level were measured. The results shown as Figure 7 indicate that, under non-heat stress conditions, compared with the control, the production rate of superoxide radicals and the hydrogen peroxide level in maize seedlings were significantly reduced by SUC alone, and the latter was also lowered by SUN + NaHS. However, NaHS alone had no significant effect on superoxide radical production and hydrogen peroxide level in maize seedlings (Figure 7A,B), while similar results related to superoxide radical production were noted in maize seedlings treated with SUC + NaHS (Figure 7A). Under heat stress conditions, superoxide radical production and hydrogen peroxide level in maize seedlings were reduced by SUC + NaHS (Figure 7A,B), and the former was also lowered by SUC alone (Figure 7A), while NaHS alone did not have a significant influence on superoxide radical production and hydrogen peroxide level (Figure 7A,B). Similarly, a significant difference in the impact of SUC alone on the hydrogen peroxide level in maize seedlings was not observed (Figure 7B).

## 3. Discussion

In plants, H_2_S and SUC are key signaling molecules which regulate many kinds of stress responses, including heat stress [2,6,7,29]. However, whether crosstalk of H_2_S and SUC signaling exists in the formation of maize thermotolerance remains unclear. Therefore, in this study, using physiological, biochemical, and molecular approaches, the metabolic and functional interactions of H_2_S with SUC in the development of thermotolerance in maize seedlings were examined (Figure 1). Their interactions are involved in the mutual regulation of the activity and gene expression of metabolic enzymes (Figure 2 and Figure 3) and antioxidant enzymes (Figure 4 and Figure 5), as well as of the content of non-enzymatic antioxidants and secondary metabolites (Figure 6). To further eliminate the effect of osmotic stress triggered by 25 mM SUC on maize thermotolerance, the seedlings were also treated with 25 mM MTL to simulate SUC treatment. The results show that, compared with the control, MTL alone had no significant effect on maize thermotolerance (Figure 1B). Also, to further investigate the specificity of SUC in maize thermotolerance, the seedlings were also treated with the inhibitor NEM of SUC transport. The results indicate that NEM alone deteriorated maize thermotolerance compared with the control (Figure 1A). These data suggest the specific effect of SUC on maize thermotolerance. 

In general, the acquisition of plant thermotolerance is an intricately physiological, biochemical, and molecular process, involving in crosstalk among signaling pathways [2,27,30,31,32]. In this study, under non-heat stress conditions, SUC treatment up-regulated the gene expression of *ZmLCD1* and *ZmOATL1* (Figure 2D,E), which in turn increased the activity of LCD, DCD, and OAS-TL (Figure 2A–C), thus accumulating endogenous H_2_S in maize seedlings (Figure 2F). Similarly, under heat stress conditions, the gene expression of *ZmLCD1* (Figure 2D), the activity of LCD and OAS-TAL (Figure 2A,C), and the endogenous H_2_S level (Figure 2F) were up-regulated by SUC. On the other hand, H_2_S treatment had no significant effect on the gene expression of *ZmSPS1* and *ZmSUS6*, the activity of SPS and SUS, or the endogenous SUC content in maize seedlings under both non-heat and heat stress conditions (Figure 3). Also, Pearson correlation analysis shows that H_2_S content displayed a significantly positive relationship with the SUC level and SUS activity (Table 1). Adversely, SUC content was also significantly positive correlated with LCD and OAS-TL activities and the endogenous H_2_S level (Table 1). These data indicate that SUC is able to trigger H_2_S signaling in maize seedlings under both non-heat and heat stress conditions, meaning that H_2_S might exert its signaling role downstream of SUC signaling.

A substantial number of studies have shown that heat stress can lead to multiple forms of damage, such as protein denaturation, biomembrane damage, osmotic stress, and oxidative stress [2,7,32]. Among these forms of damage, oxidative stress is a major determinant, which can further cause other damages [2,27,32,33]. Therefore, the acquisition of plant thermotolerance is closely associated with the mitigation of oxidative stress [16,29,34,35,36,37]. In this study, to further explore the underlying mechanism of SUC–H_2_S interaction-promoted thermotolerance in maize seedlings, antioxidant enzymes, non-enzymatic antioxidants, and secondary metabolites were analyzed. Under non-heat stress conditions, compared with the control, SUC treatment increased CAT and GR activities (Figure 4A,B), up-regulated *ZmDHAR1* and *ZmMDHAR1* expression (Figure 5A,B), and accumulated flavone and carotenoids (Figure 6B,D), thus reducing the production rate of superoxide radicals in maize seedlings (Figure 7A). Similarly, CAT activity (Figure 4A), *ZmMDHAR1* expression (Figure 5B), and AsA and flavone levels (Figure 6A,D) in maize seedlings were elevated by H_2_S. Also, MDHAR activity, *ZmMDHAR1* expression, and AsA, flavone, and carotenoid contents were promoted by SUC in combination with H_2_S in maize seedlings. Moreover, under non-stress conditions, exogenous SUS treatment could enhance the antioxidant system composed of antioxidant enzymes (POD, CAT, APX, GR, and SOD) and non-enzymatic antioxidants (AsA, glutathione, and anthocyanin) [12,13]. Taken together, before heat stress, the antioxidant enzymes, non-enzymatic antioxidants, and secondary metabolites enhanced by SUC and H_2_S alone or in combination lay the physiological, biochemical, and molecular foundations for the development of subsequent thermotolerance in maize seedlings.

Under heat stress conditions, *ZmMDHAR1* gene expression (Figure 5B) as well as AsA and flavone contents (Figure 6A,D) in maize seedlings were increased by SUC, which in turn decreased the production rate of superoxide radicals (Figure 6C). Analogously, the GR and MDHAR activity (Figure 4B and Figure 5B), *ZmMDHAR1* expression (Figure 5B), and AsA content (Figure 6A) in maize seedlings were augmented by H_2_S. Moreover, SUN in combination with H_2_S up-regulated *ZmMDHAR1* expression (Figure 5B) and increased AsA and carotenoid contents (Figure 6A,B) in maize seedlings, thus decreasing superoxide radical production (Figure 6C) and hydrogen peroxide accumulation (Figure 7A,B). In addition, Pearson correlation analysis indicates that the survival rate was markedly positively correlated with CAT, GR, DHAR, and MDHAR activities (Table 2). Similarly, the survival rate displayed a significant positive relationship with AsA, flavone, carotenoid, and hydrogen peroxide levels, while it displayed a negative correlation with the production of superoxide radicals (Table 3). Also, under heat stress conditions, exogenous SUC treatment increased the endogenous SUC level in maize seedlings (Figure 3C). An increased SUC level had multiple functions, including as a osmoprotectant, an ROS-scavenger, and small chaperone [15,16,17], thus alleviating oxidative damage and osmotic stress induced by heat stress. These data imply that the maize seedlings treated with SUC and H_2_S alone or in combination maintained a high antioxidant capacity under heat stress conditions, thus reducing oxidative damage and improving maize thermotolerance (Figure 1). In rice seedlings, foliage sprayed with SUC displayed increased CAT activity, as well as SUC, total soluble sugar, and NAD^+^ contents, which in turn reduced MDA and hydrogen peroxide accumulation, thus improving photosynthetic efficiency and increasing thermotolerance in rice seedlings [18]. Also, in potato plants, SUC treatment increased SOD, POD, CAT, and APX activities, as well as SUC, total sugar, proline, and soluble protein contents, followed by a decrease in hydrogen peroxide level, thus alleviating oxidative damage and osmotic stress caused by heat stress [19]. Also, in Arabidopsis plants, SUC, as a signaling molecule, can induce the gene expression of heat shock proteins via the target of the rapamycin-E2F (transcription factor) signaling module, thus governing thermomemory and improving plant thermotolerance [38,39]. These studies further support our current hypothesis that the metabolic and functional interactions of H_2_S and SUC exist in the formation of maize thermotolerance through redox homeodynamics.

## 4. Materials and Methods

### 4.1. Seed Germination and Seedling Treatment

In this work, maize (*Zea mays* L., cv. Diyu No. 401) seeds were purchased from Diyu Seed Company, China, and then immersed in 5% sodium hypochlorite (NaClO) solution for 10 min for sterilization. The sterilized seeds were imbibed in distilled water at 26 °C for 12 h after washing. The imbibed seeds were germinated on eight-layer filter paper (which was wetted with distilled water) in trays with covers (approximately 250 seeds per tray) at 26 °C for 60 h (namely 2.5 d). After germination, the 2.5 d old seedlings were classified into 11 groups, which were irrigated with 90 mL of solutions listed as follows for 12 h, respectively: (1) distilled water (control, CK); (2) 500 μM NaHS (NaHS); (3) 25 mM sucrose (SUC); (4) 25 mM mannitol (MTL); (5) 300 μM N-ethylmaleimide (NEM); (6) 300 μM NEM + 500 μM NaHS (NEM + NaHS); (7) 25 mM SUC + 500 μM NaHS (SUC + NaHS); (8) 300 μM propargylglycine + 25 mM SUC (PAG + SUC); (9) 300 μM hydroxylamine + 25 mM SUC (HA + SUC); (10) 300 μM PAG (PAG); and (11) 300 μM HT (HT).

Based on the previous reports, NaHS, PAG, and HT are a donor, inhibitor, and scavenger of H_2_S [40,41], respectively, while NEM is a SUC transport inhibitor [42]. An appropriate concentration of NaHS, SUC, MTL, and other pharmacological reagents was selected from the preliminary experiments and previous reports [40,43,44]. After treatment with the reagents for 12 h, the treated seedlings were subjected to heat stress at 46 °C for 16 h. After heat stress, the seedling mesocotyls (the most sensitive organ to heat stress [45]) were cut and used to estimate the following physiological and molecular parameters.

### 4.2. Estimation of Thermotolerance Indexes

To illustrate the effect of H_2_S–SUC interaction on maize thermotolerance, after heat stress and recovery at 26 °C for a week, the survival rate (%) was calculated as per the ratio of the number of surviving seedlings to total number of seedlings. Also, before and after heat stress, tissue viability, malondialdehyde (MDA) content, and electrolyte leakage in the mesocotyls of maize seedlings were estimated as per the report by Wang et al. [7]. The electrolyte leakage was indicated by %, whereas MDA content and tissue viability were expressed in nmol g^−1^ fresh weight (FW) and A_485_, respectively.

### 4.3. Measurement of Metabolizing Enzymes and Endogenous Content of H_2_S

To study the effect of SUC on the metabolizing enzyme activity and endogenous level of H_2_S, the maize seedlings were irrigated with SUC and NaHS alone or in combination and then exposed to heat stress. For the analysis of enzyme activity, the mesocotyls (0.2 g) were ground with a mortar and pestle in liquid nitrogen, and then 2 mL of 20 mM Tris–HCl (pH 8.0) was added to homogenates to extract LCD, DCD, and OAS-TL. After centrifugation at 10,000× *g* for 10 min at 4 °C, the enzyme activity was measured as per the methylene blue method [8,45]. Similarly, H_2_S in mesocotyls was extracted in 2 mL of 50 mM phosphate buffer (pH 6.8) containing 0.1 mM EDTA and 0.2 mM AsA. The homogenate was used to assay for H_2_S content in light of methylene blue method [8,45]. Enzyme activity was indicated by nmol min^−1^ g^−1^ FW, while H_2_S content was expressed in μmol g^−1^ FW. Also, the gene expression of *ZmLCD1* and *ZmOAS-TL* was quantified by qRT-PCR, and *Zea mays* beta-5 tubulin (*ZmTUB*) was used as an internal reference. The expression level was counted using 2^−∆∆CT^ [7], and gene primers are listed in Supplement S1.

### 4.4. Analysis of Metabolizing Enzymes and Endogenous Content of SUC

Also, to investigate the effect of H_2_S on the metabolizing enzyme activity and endogenous content of SUC, which were analyzed after the maize seedlings were irrigated with SUC and NaHS alone or in combination and subsequently subjected to heat stress, the extraction and analysis of SPS, SUS, and SUC in mesocotyls of maize seedlings referred to the procedures of Zhu et al. [46]. The SUC level and SPS activity were analyzed using resorcinol methods, and the latter was determined by measuring the formation of sucrose (plus sucrose-6-P) in the reaction mixture. Also, the analysis of SUS activity was in accordance with SPS, except that pH in the reaction buffer was changed to 8.5 and fructose was replaced with fructose-6-P. The enzyme activity and SUC content were expressed in mg min^−1^ g^−1^ FW and mg g^−1^ FW, respectively. Also, the gene expression of *ZmSPS1* and *ZmSUS6* was quantified and calculated according to the procedures mentioned above.

### 4.5. Evaluation of Antioxidant Capacity

To further establish the underlying mechanism of H_2_S–SUC interaction-promoted maize thermotolerance, antioxidant enzymes, non-enzymatic antioxidants, and secondary metabolites were evaluated before and after heat stress. Antioxidant enzymes in mesocotyls were extracted in 2 mL of 50 mM Tris-HCl (pH 7.0) containing 0.1 mM EDTA, 1 mM AsA, 1 mM dithiothreitol (DTT), and 5 mM MgCl_2_. The homogenates were centrifuged at 10,000× *g* for 15 min at 4 °C, and the supernatants were used to measure CAT, GR, APX, POD, DHAR, MDHAR, and SOD activities according to the methods of Wang et al. [7] and Sun et al. [45]. An extinction coefficient (40 nm mM^−1^ cm^−1^ at 240 nm, 6.2 nm mM^−1^ cm^−1^ at 340 nm, 2.8 nm mM^−1^ cm^−1^ at 290 nm, 26.6 nm mM^−1^ cm^−1^ at 470 nm, 14.0 nm mM^−1^ cm^−1^ at 265 nm, 14.0 nm mM^−1^ cm^−1^ at 265 nm, and 21.6 nm mM^−1^ cm^−1^ at 560 nm) was used to calculate the activity of corresponding enzymes (CAT, GR, APX, POD, DHAR, MDHAR, and SOD, respectively). Enzyme activity was expressed in nmol min^−1^ g^−1^ FW.

Also, the gene expression of *ZmCAT1*, *ZmGR1*, *ZmDHAR1*, *ZmMDHAR1*, and *ZmSOD4* was quantified and calculated according to the methods stated above. In addition, the ascorbic acid (AsA), flavonone, carotenoid, and total phenol contents in the mesocotyls of maize seedlings were evaluated based on the report by Wang et al. [7]. These contents were expressed in nmol g^−1^ FW and μg g^−1^ FW, respectively.

### 4.6. Assay for ROS

To explore the effect of H_2_S–SUC interaction on the production rate of superoxide radicals (O_2_^•−^) and the hydrogen peroxide (H_2_O_2_) level in the mesocotyls of maize seedlings, before and after heat stress, their production and level were assayed. Superoxide radical (O_2_^•−^) production and hydrogen peroxide (H_2_O_2_) level were separately determined using the Na,3-[1-[(phenylamino)-carbonyl]-3,4-tetrazolium] (4-methoxy-6-nitro) benzene sulfonic acid hydrate method [45] and the titanium sulfate method [7]. An extinction coefficient (21.6 mM^−1^ cm^−1^ at 470 nm and 0.28 mM^−1^ cm^−1^ at 410 nm) was used to calculate superoxide radical production and the hydrogen peroxide level, which were expressed in nmol min^−1^ g^−1^ FW and nmol g^−1^ FW, respectively. 

### 4.7. Statistical Analysis

Experiments were carried out based on a random design and at least three biological replicates were used. The data analysis was executed as per one-way analysis of variance (ANOVA), and Duncan’s multiple-range test was used to estimate significant differences among data. In the figures, the same letters on the bars denote no significant difference, whereas different letters denote significant differences. The data in the figures indicate means ± standard errors (SEs). In addition, Pearson correlation analysis was performed using sigmaplot 15, and the asterisks (*) and double asterisks (**) in the tables denote significant (*p* ˂ 0.05) and very significant (*p* ˂ 0.01) differences.

## 5. Conclusions

**Figure 8:**
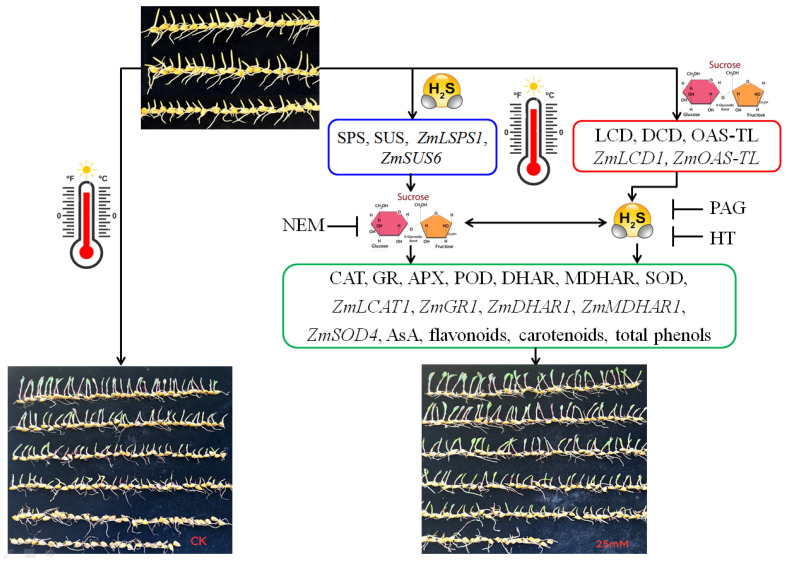
The underlying mechanism of hydrogen sulfide (H_2_S) and sucrose interaction-promoted maize thermotolerance. H_2_S signaling could be triggered by exogenous sucrose via activating L-cysteine desulfhydrase (LCD), D-cysteine desulfhydrase (DCD), O-acetyl-serine (thiol) lyase (OAS-TL) activities and the corresponding gene expression of *ZmLCD1* and *ZmOAS-TL*. Also, sucrose signaling could be modulated by H_2_S via regulating sucrose-P synthase (SPS) and sucrose synthase (SUS) activities and the corresponding gene expression of *ZmSPS1* and *ZmSUS6*. Therefore, the interaction of H_2_S and sucrose signaling promoted maize thermotolerance by enhancing antioxidant enzymes, non-enzymatic antioxidants, and secondary metabolites.

Taken together, the findings of this paper are summarized in Figure 8. It is clear that heat stress leads to a decrease in the survival rate of maize seedlings under heat stress. However, SUC and H_2_S alone or in combination increased the survival rate of maize seedlings under heat stress conditions, and the SUC-promoted survival rate was weakened by a SUC-transport inhibitor (NEM) and an inhibitor (PAG) and scavenger (HT) of H_2_S, indicating the interaction of H_2_S and SUC in the development of maize thermotolerance. Further, SUC treatment, to different extents, up-regulated LCD, DCD, and OAS-TL activities and the corresponding gene expression, as well as the endogenous H_2_S level in maize seedlings under non-heat and heat stress conditions, further supporting the fact that H_2_S interacted with SUC. Also, SUC and H_2_S alone or in combination, to varying degrees, enhanced antioxidant enzyme activity and their gene expression, as well as the non-enzymatic antioxidant level including secondary metabolites in maize seedlings under non-heat and heat stress conditions, suggesting the functional interactions of H_2_S and SUC in maize thermotolerance. These data illustrate that the metabolic and functional interactions of H_2_S and SUC exist in the formation of maize thermotolerance via cellular redox homeodynamics. This finding lays out the physiological and molecular basis for developing climate-resistant maize crops and promoting sustainable agriculture.

## Figures and Tables

**Table 1:** Pearson correlation analysis between H_2_S and its metabolic enzymes and SUC and its metabolic enzymes including L-cysteine desulfhydrase (LCD), D-cysteine desulfhydrase (DCD), O-acetyl-serine (thiol) lyase (OAS-TL), sucrose (SUC), sucrose-P synthase (SPS), and sucrose synthase (SUS). R (numbers in table) denotes the correlation coefficient, whereas asterisks (*, *p* < 0.05) and double asterisks (**, *p* < 0.05) indicate significant and very significant differences.

r	H_2_S	LCD	DCD	OAS-TL
SUC	0.712 *	0.804 **	0.126	0.421 *
SPS	0.520	0.623 *	0.201	0.512 *
SUS	0.651	0.530 *	0.321	0.450 *

**Table 2:** Pearson correlation analysis among survival rate, catalase (CAT), glutathione reductase (GR), ascorbate peroxidase (APX), peroxidase (POD), dehydroascorbate reductase (DHAR), monodehydroascorbate reductase (MDHAR), and superoxide dismutase (SOD). R (numbers in table) denotes correlation coefficient, whereas asterisks (*, *p* < 0.05) and double asterisks (**, *p* < 0.05) indicate significant and very significant differences.

r	Survival Rate	CAT	GR	APX	POD	DHAR	MDHAR	SOD
Survival rate	1							
CAT	0.731 **	1						
GR	0.654 **	0.320 *	1					
APX	0.321	0.412	0.437 *	1				
POD	0.452	0.214	0.213	0.234	1			
DHAR	0.641 **	0.426 *	0.512 *	0.423 *	0.254	1		
MDHAR	0.536 *	0.402 **	0.342 *	0.312 *	0.315	0.452 *	1	
SOD	0.132	0.204	0.206	0.217	0.218	0.256	0.235	1

**Table 3:** Pearson correlation analysis among survival rate, ascorbic acid (AsA), flavone, carotenoid, total phenol, hydrogen peroxide (H_2_O_2_), and superoxide radical (O_2_^•−^). R (numbers in table) denotes correlation coefficient, whereas asterisk (*, *p* < 0.05) indicate significant.

r	Survival Rate	AsA	Flavone	Carotenoid	Total Phenol	H_2_O_2_	O_2_^•−^
Survival Rate	1						
AsA	0.601 *	1					
Flavone	0.352 *	0.321	1				
Carotenoid	0.314 *	0.432 *	0.410 *	1			
Total Phenol	0.215	0.284	0.256 *	0.413 *	1		
H_2_O_2_	0.412 *	0.402 *	0.426 *	−0.246 *	0.622 *	1	
O_2_^•−^	−0.523 *	−0.346	−0.532 *	−0.420 *	−0.516 *	−0.421 *	1

## Data Availability

All data are displayed in the manuscript and Supplementary Materials.

## Supplementary Materials

The following supporting information can be downloaded at: https://www.mdpi.com/article/10.3390/ijms25126598/s1.
