# Supplementary material for: Metabolic and Functional Interactions of H2S and Sucrose in Maize Thermotolerance through Redox Homeodynamics
Source: Int J Mol Sci. 2024 Jun 15;25(12):6598. doi: 10.3390/ijms25126598 (PMC11204011; doi:10.3390/ijms25126598)
Supplement: Supplementary file 1 [file ijms-25-06598-s001.zip › ijms-3040557-supplementary.pdf]

Supplement Table S1 Genes and their primers in this study.

Gene Name	Access Number	Sequence Forward （5'—3'）
ZmTUB	NM_001111988	F:AGAACTGCGACTGCCTCCAAAGG
		R:AGATGAGCAGGGTGCCCATTC
ZmLCD1	NM_001138259	F:AAGTGTTGAGGAAGGACAAGAG
		R:GGCATCTCTCAAGACCTCATAC
ZmOAS-TL	NM_001366967	F:GGCAAGTACCTCAAGGAGAAA
		R:CTACTCCGTTTCCAGTGATGAG
ZmSUS6	XM_008680885.2	F:CTGATAGGTCGGCAACAAGG
		R:TCGGGCACACATCTGGTT
ZmSPS1	NM_001112224.2	F:AGAAGGGTTCGGAAGCACTG
		R:CCGCGGTACTGTTCACAAC
ZmCAT1	NM_001254879.2	F:GGGTCCAGACACCTGTTATTG
		R:AGTTACCCTCTCTGGTGTAGAA
ZmSOD4	NM_001112234.2	F:CGTCACCAGCAGGCTAGAAT
		R:AGCCAACAGTCCAACACAGT
ZmGR1	NM_001305818.1	F:CTCTCACGAGTTTGAAGAGTCTCGTGG
		R:CCAGCGCAGCATCCGAATCTATAA
ZmAPX1	NM_001370758.1	F:GATCTTGTGGCTGCAGCATG
		R:GGTGGACTCGAATTGCAGGA
ZmMDHAR	NM_001196274.1	F:AAGTGGTGGAGAGAAGCTATTG
		R:CTAGTCAGAGTCTTGGTGGAAAG
ZmDHAR1	NM_001147572.1	F:ATCTCTGGTCACTCCTGTAGAA
		R:CTCGGAACCATCACTAGCATC
